# Acute and Subacute Toxicity *In Vivo* of Thermal-Sprayed Silver Containing Hydroxyapatite Coating in Rat Tibia

**DOI:** 10.1155/2014/902343

**Published:** 2014-03-20

**Authors:** Masatsugu Tsukamoto, Hiroshi Miyamoto, Yoshiki Ando, Iwao Noda, Shuichi Eto, Takayuki Akiyama, Yutaka Yonekura, Motoki Sonohata, Masaaki Mawatari

**Affiliations:** ^1^Department of Orthopedic Surgery, Faculty of Medicine, Saga University, 5-1-1 Nabeshima, Saga 849-8501, Japan; ^2^Departments of Pathology and Microbiology, Faculty of Medicine, Saga University, Saga 849-8501, Japan; ^3^Research Department, KYOCERA Medical Corporation, Osaka 532-0003, Japan

## Abstract

To reduce the incidence of implant-associated infection, we previously developed a novel coating technology using hydroxyapatite (HA) containing silver (Ag). This study examined *in vivo* acute and subacute toxicity associated with the Ag-HA coating in rat tibiae. Ten-week-old rats received implantation of HA-, 2% Ag-HA-, or 50% Ag-HA-coated titanium rods. Concentrations of silver in serum, brain, liver, kidneys, and spleen were measured in the acute phase (2–4 days after treatment) and subacute phase (4–12 weeks after treatment). Biochemical and histological examinations of those organs were also performed. Mean serum silver concentration peaked in the acute phase and then gradually decreased. Mean silver concentrations in all examined organs from the 2% Ag-HA coating groups showed no significant differences compared with the HA coating group. No significant differences in mean levels of glutamic-oxaloacetic transaminase, glutamic-pyruvic transaminase, lactate dehydrogenase, creatinine, or blood urea nitrogen were seen between the three groups and controls. Histological examinations of all organs revealed no abnormal pathologic findings. No acute or subacute toxicity was seen *in vivo* for 2% Ag-HA coating or HA coating. Ag-HA coatings on implants may represent biologically safe antibacterial biomaterials and may be of value for reducing surgical-site infections related to implantation.

## 1. Introduction

Orthopedic implants are widely used in many countries to provide pain relief and improve range of motion and quality of life for patients with arthritic disorder. However, one of the serious postoperative complications associated with these procedures is bacterial infection, including surgical-site infection (SSI). The rate of primary infection for joint replacement is between 0.86% and 2.52% according to the National Nosocomial Infections Surveillance System [1]. Infections that occur following implantation may require long-term treatment, including replacement of the infected artificial joint, resection arthroplasty, or amputation, depending on the severity of symptoms.

One way to reduce the incidence of implant-related infection is to use implants possessing innate antibacterial properties. Various antibacterial coatings, such as vancomycin [2], gentamicin [3], carbonated hydroxyapatite (HA) [4], nitric oxide-releasing xerogel [5], and iodine [6, 7], have been developed for use on implant surfaces. We previously developed a novel coating technology composed of HA containing silver (Ag) to reduce the incidence of implant-associated infections and have reported that this coating has the properties of HA, induces the release of silver ions [8], and shows high antibacterial activity with inhibition of bacterial adhesion and low cytotoxicity* in vitro* [9]. In addition, using rat subcutaneous [10] and tibial implantations [11], we demonstrated that the Ag-HA coating possesses antibacterial activity against methicillin-resistant* Staphylococcus aureus in vivo*. Furthermore, the 3% Ag-HA coating has been shown to provide good osteoconductivity and has been comparable with an HA coating and a 50% Ag-HA coating in the inhibition of bone formation because silver toxicity depended on the dose [12]. Therefore, the appropriate silver concentrations of Ag-HA are around 2-3%. However, data on local and systemic toxicity of the Ag-HA coating remain scarce. Before Ag-HA-coated implants can be applied clinically, evaluation of the risk of silver toxicity* in vivo* is required.

The aim of this study was to clarify local and systemic adverse effects of Ag-HA-coated implants in rat tibiae.

## 2. Materials and Methods

### 2.1. Coating and Implants

The implants used were Ti wire 20 mm in length and 1 mm in diameter (HOMS, Nagano, Japan). Ag_2_O powder (Kanto Chemical, Tokyo, Japan) was mixed with HA powder (KYOCERA Medical Materials, Osaka, Japan) in concentrations of 2% and 50% and then shaken for 5 min in a plastic bag. The mix was then sprayed onto the surface of the Ti wire using the Frame Sprayed System (Sulzer Metco Japan, Tokyo, Japan), which uses an acetylene torch. Temperature of the flame was about 2700°C. This procedure was performed under normal atmosphere. The properties of this coating have been reported by Akiyama et al. [11]. In brief, a 40 ± 10 *μ*m thick layer of Ag-HA was uniformly coated on the substrate. Amorphous structure of the Ag-HA coating was confirmed for concentrations ≤50% Ag. Qualitative analysis revealed that HA and Ag-HA coatings contained similar levels of Ca and P, although Ag was not identified. According to quantitative elementary analysis, the Ca/P ratio of the Ag-HA coating (1.74) was almost the same as that of the HA coating (1.67).

We prepared three types of implant as follows: (i) HA coating on Ti; (ii) 2% Ag-HA coating on Ti; and (iii) 50% Ag-HA coating on Ti. Implants were packaged singly and sterilized using a JS-8500 gamma sterilizer (MDS Nordion, Ontario, Canada). The implants were obtained from KYOCERA Medical Materials Corporation (Osaka, Japan).

### 2.2. Animals

We used 10-week-old male Sprague-Dawley rats (Kyudo, Saga, Japan) with a mean weight of 382.5 ± 13.7 g. Rats were acclimatized for 5 days prior to use, in a room in which a suitable environment was maintained. All animal procedures were conducted with the approval of the Animal Research Ethics Committee at Saga University (approval number: 23-009-1). According to their recommendation that the number of experimental animals be kept as low as possible, the sample was limited to three or six rats per group, as the minimum number needed to calculate the mean and standard deviation.

### 2.3. Operative Technique

Rats were anesthetized using a 50 mg/kg intraperitoneal injection of pentobarbital sodium solution prior to the surgical procedure. Both hind legs were shaved, cleaned with povidone-iodine, and dried. One transverse 10 mm incision was made on both knees. To access the medullary cavity, a hole was drilled with an 18-gauge needle through tibial tuberosity, and the same implants were inserted into the medullary cavity of both tibiae in each rat. Rats were divided into three groups according to the implant inserted. They were housed individually with* ad libitum* access to food and water. Clinical parameters and general condition were documented weekly. Body weight was measured after 2, 4, 6, 8, 10, and 12 weeks. Three rats from each group were euthanized at 2, 3, and 4 days after treatment. Six rats from each group were euthanized at 4, 8, and 12 weeks after treatment. The acute phase was defined as 2–4 days after treatment, and the subacute phase was defined as 4–12 weeks after treatment.

The abdomen was then opened under general anesthesia and 10 mL of blood was collected from the right common iliac vein. The liver, kidneys, spleen, and brain were obtained after euthanasia. Postmortem examinations included macroscopic inspection of the skin after shaving. The inspection focused on the presence of ash-colored skin, which can occur in argyria (systemic silver intoxication).

### 2.4. Histological Examination

Histological examinations used nine rats from each group, with three rats each euthanized at 4, 8, and 12 weeks postoperatively. Histological samples were obtained from the brain, liver, kidneys, and spleen. These organs were fixed in 10% phosphate-buffered formaldehyde and embedded in paraffin wax. Sections of ~5 *μ*m were stained with hematoxylin and eosin. Stained sections from each test sample were then examined under light microscopy for analysis of tissue inflammatory reaction, fibrosis, and the presence of silver sedimentation.

### 2.5. Blood Serum Analysis

The concentration of silver in serum was determined by inductively coupled plasma-mass spectrometric analysis (ICP-MS) (Perkin Elmer, Waltham, MA). In addition, the following parameters were determined: glutamic-oxaloacetic transaminase (GOT); glutamic-pyruvic transaminase (GPT); lactate dehydrogenase (LDH); blood urea nitrogen (BUN); and creatinine. Since no standard values were available for GOT, GPT, LDH, BUN, and creatinine for 10-, 14-, 18-, and 22-week-old Sprague-Dawley rats, these values were determined preoperatively in 20 healthy rats. Standard values were assumed if they ranged between 2.5% and 97.5% percentiles.

### 2.6. Organ Analysis

Organ analysis involved the measurement of concentrations of silver in the brain, liver, kidneys, and spleen. Three rats from each group were euthanized for organ analysis at 2, 3, and 4 days and 4, 8, and 12 weeks postoperatively. These organs were obtained from rats and washed using ultrapure water provided by a Milli-Q water purification system (Millipore, Bedford, MA). Concentrations of silver in the brain, liver, kidneys, and spleen were measured using an Agilent 7500c inductively coupled plasma-mass spectrometer (ICP-MS) (Agilent, Santa Clara, CA).

### 2.7. Statistical Analysis

This was performed using Excel and SPSS version 19 software (SPSS, Chicago, IL). One-way analysis of variance was used to evaluate body weight, blood serum analysis, and organ analysis. Where this was significant, Tukey's honestly significant difference (HSD) was applied as a* post hoc* test. Values of *P* ≤ 0.05 were considered significant.

## 3. Results

### 3.1. Body Weight, General Condition, and Skin Condition after Implanting

**Figure 1:**
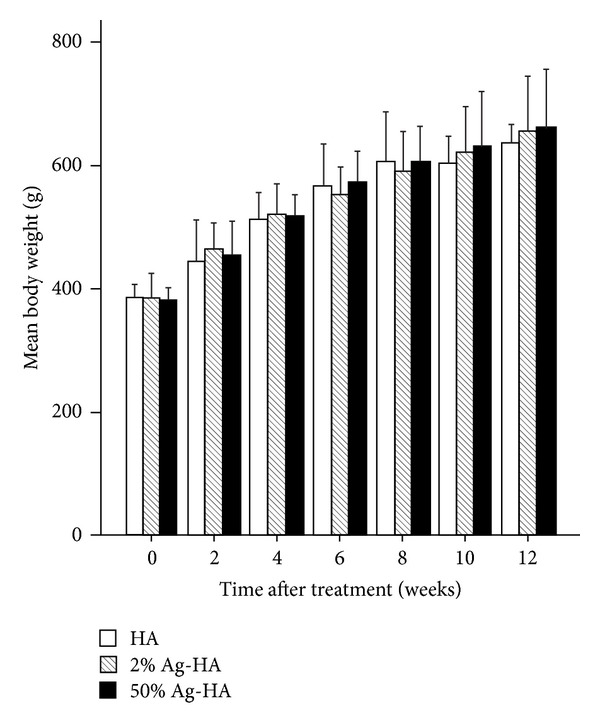
Mean body weight at 2, 4, 6, 8, 10, and 12 weeks after treatment in the hydroxyapatite (HA), 2% Ag-HA, and 50% Ag-HA coating groups. No significant differences were evident between the three groups for all experimental periods.

Mean body weights preoperatively in the HA, 2% Ag-HA coating, and 50% Ag-HA coating groups were 383.1 ± 10.6 g, 382.9 ± 16.4 g, and 381.7 ± 13.0 g, respectively (Figure 1). Mean body weights at 12 weeks in the HA, 2% Ag-HA, and 50% Ag-HA coating groups were 634.7 ± 15.4 g, 656.4 ± 50.5 g, and 683.3 ± 88.3 g, respectively. No significant differences in mean body weight were found between the three groups at 2, 4, 6, 8, 10, and 12 weeks.

No animals in any of the three groups died during the measurement period. No animals showed any signs of SSI. No animals displayed any signs of local or systemic argyrosis (data not shown).

### 3.2. Serum Analysis

**Figure 2:**
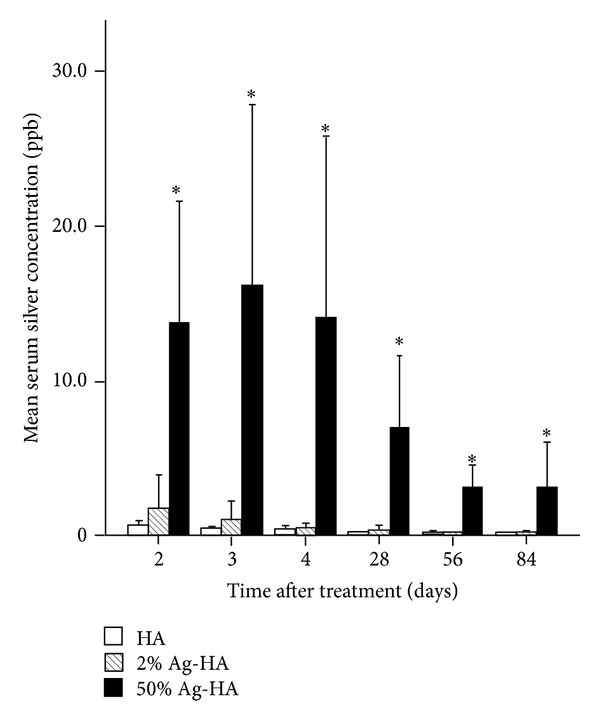
Mean serum silver concentration at 2, 3, 4, 28, 56, and 84 days after treatment in the hydroxyapatite (HA), 2% Ag-HA, and 50% Ag-HA coating groups. *Significant difference between the 50% Ag-HA coating group and the other two groups for all experimental periods (*P* < 0.01).

The HA, 2% Ag-HA, and 50% Ag-HA coating groups showed mean serum silver concentrations of 0.60 ± 0.17 ppb, 1.75 ± 1.08 ppb, and 13.8 ± 3.95 ppb at 2 days, respectively, and 0.37 ± 0.06 ppb, 0.95 ± 0.63 ppb, and 16.2 ± 5.85 ppb at 3 days, respectively (Figure 2). The silver concentration of serum decreased gradually over the experimental period. Mean concentration of silver in the 50% Ag-HA coating group differed significantly from that in the other two groups at acute and subacute phase (*P* < 0.01 for all comparisons at all periods, Tukey's HSD test). Mean concentration of silver in the 2% Ag-HA coating group showed no significant difference compared with the HA coating group in any experimental periods.

Liver function was assessed by determination of GOT, GPT, and LDH levels. Kidney function was assessed by measurement of creatinine and BUN levels.

Mean GOT, GPT, LDH, creatinine, and BUN of the HA, 2% Ag-HA, and 50% Ag-HA coating groups were not significantly higher than in controls at 2, 3, and 4 days (Table 1).

At 4 weeks, mean GOT differed significantly between the control and HA coating groups, but mean GPT and LDH in the HA coating group were not significantly different from that in the control group (Table 2). No significant difference between the control, 2% Ag-HA, and 50% Ag-HA coating groups was noted for any laboratory parameters. At 8 weeks, mean GOT and LDH differed significantly between the control and HA coating groups. No significant differences were found between mean GPT in the control and HA coating groups. No significant differences were seen between the control, 2% Ag-HA, and 50% Ag-HA coating groups for any laboratory parameters. At 12 weeks, no significant differences were seen between the control, HA, 2% Ag-HA, and 50% Ag-HA coating groups for any laboratory parameters.

### 3.3. Organ Analysis

#### 3.3.1. Brain

**Figure 3:**
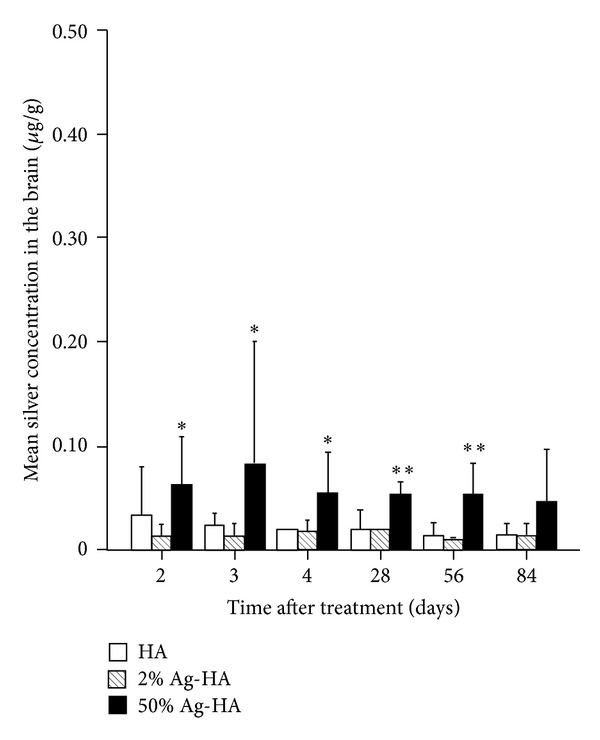
Mean silver concentration in the brain at 2, 3, 4, 28, 56, and 84 days after treatment in the hydroxyapatite (HA), 2% Ag-HA, and 50% Ag-HA coating groups. *Significant differences between the 50% Ag-HA coating group and the other two groups at 2, 3, and 4 days (*P* < 0.05). **Significant differences between the 50% Ag-HA coating group and the other two groups at 28 and 56 days (*P* < 0.01).

Mean silver concentrations in the brain for the HA, 2% Ag-HA, and 50% Ag-HA coating groups at 2 days were 0.02 ± 0.00 *μ*g/g, 0.01 ± 0.01 *μ*g/g, and 0.06 ± 0.02 *μ*g/g, respectively (Figure 3). At 3 days, mean silver concentrations in the brain for HA, 2% Ag-HA, and 50% Ag-HA coating groups were 0.02 ± 0.01 *μ*g/g, 0.01 ± 0.01 *μ*g/g, and 0.05 ± 0.01 *μ*g/g, respectively. At 4 days, mean silver concentrations in the brain for HA, 2% Ag-HA, and 50% Ag-HA coating groups were 0.02 ± 0.00 *μ*g/g, 0.02 ± 0.01 *μ*g/g, and 0.05 ± 0.02 *μ*g/g, respectively. The value in the 50% Ag-HA coating group was significantly different from that in the HA and 2% Ag-HA coating groups at 2, 3, and 4 days (*P* < 0.05 for all comparisons at all periods, Tukey's HSD test). No significant difference was apparent between HA and 2% Ag-HA coating groups at 2, 3, and 4 days.

Furthermore, mean silver concentrations in the brain for HA, 2% Ag-HA, and 50% Ag-HA coating groups at 4 weeks were 0.02 ± 0.01 *μ*g/g, 0.02 ± 0.00 *μ*g/g, and 0.05 ± 0.01 *μ*g/g, respectively. At 8 weeks, mean silver concentrations in the brain for HA, 2% Ag-HA, and 50% Ag-HA coating groups were 0.01 ± 0.01 *μ*g/g, 0.01 ± 0.00 *μ*g/g, and 0.05 ± 0.02 *μ*g/g, respectively. The values in the 50% Ag-HA coating group differed significantly from those in the HA and 2% Ag-HA coating groups at 4 and 8 weeks (*P* < 0.01 for all comparisons at all periods, Tukey's HSD test). No significant differences were found between the HA and 2% Ag-HA coating groups at 4 and 8 weeks. At 12 weeks, mean silver concentrations in the brain for HA, 2% Ag-HA, and 50% Ag-HA coating groups were 0.01 ± 0.01 *μ*g/g, 0.01 ± 0.01 *μ*g/g, and 0.05 ± 0.03 *μ*g/g, respectively. No significant differences were found between the three groups.

#### 3.3.2. Liver

**Figure 4:**
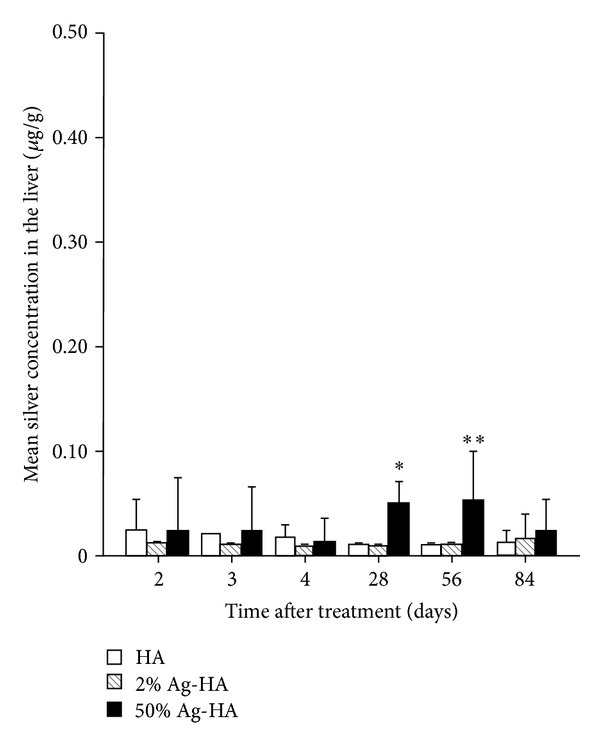
Mean silver concentration in the liver at 2, 3, 4, 28, 56, and 84 days after treatment in the hydroxyapatite (HA), 2% Ag-HA, and 50% Ag-HA coating groups. *Significant difference between the 50% Ag-HA coating group and the other two groups at 28 days (*P* < 0.01). **Significant differences between the 50% Ag-HA coating group and the other two groups at 56 days (*P* < 0.05).

Mean silver concentrations in the liver for the HA, 2% Ag-HA, and 50% Ag-HA coating groups at 2 days were 0.02 ± 0.01 *μ*g/g, 0.01 ± 0.00 *μ*g/g, and 0.04 ± 0.02 *μ*g/g, respectively (Figure 4). At 3 days, mean silver concentrations for the HA, 2% Ag-HA, and 50% Ag-HA coating groups were 0.02 ± 0.00 *μ*g/g, 0.01 ± 0.00 *μ*g/g, and 0.04 ± 0.01 *μ*g/g, respectively. At 4 days, mean silver concentrations for the HA, 2% Ag-HA, and 50% Ag-HA coating groups were 0.02 ± 0.01 *μ*g/g, 0.01 ± 0.00 *μ*g/g, and 0.02 ± 0.00 *μ*g/g, respectively. No significant differences were found between the three groups at 2, 3, or 4 days.

Mean silver concentrations in the liver for the HA and 2% Ag-HA coating groups were 0.01 ± 0.00 *μ*g/g at 4 and 8 weeks. Mean silver concentrations in the liver for the 50% Ag-HA coating group at 4 and 8 weeks were 0.05 ± 0.01 *μ*g/g and 0.05 ± 0.02 *μ*g/g, respectively. The value in the 50% Ag-HA coating group differed significantly from those in the HA and 2% Ag-HA coating groups (*P* < 0.01 for all comparisons at 4 weeks; *P* < 0.05 for all comparisons at 8 weeks, Tukey's HSD test). No significant differences between the HA and 2% Ag-HA coating groups were seen at 4 and 8 weeks. At 12 weeks, mean silver concentrations in the HA, 2% Ag-HA, and 50% Ag-HA coating groups were 0.01 ± 0.01 *μ*g/g, 0.02 ± 0.01 *μ*g/g, and 0.02 ± 0.02 *μ*g/g, respectively. No significant differences were found between the three groups.

#### 3.3.3. Kidney

**Figure 5:**
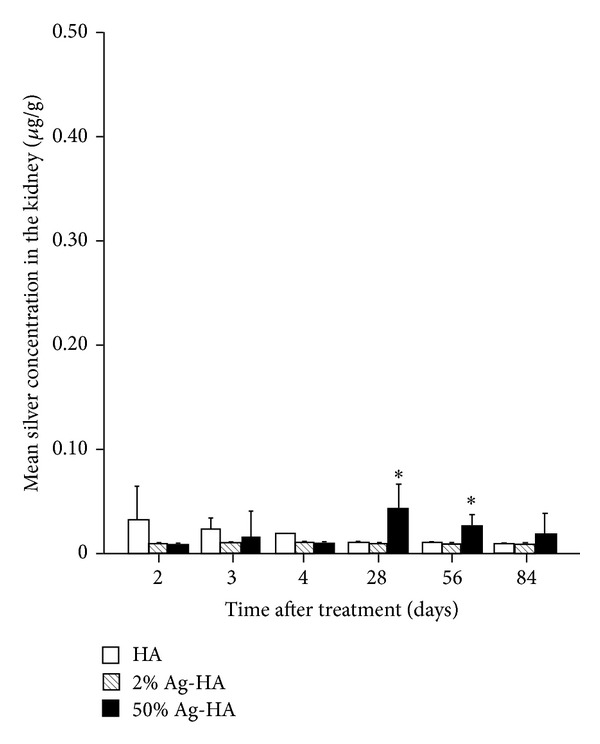
Mean silver concentration in the kidney at 2, 3, 4, 28, 56, and 84 days after treatment in the hydroxyapatite (HA), 2% Ag-HA, and 50% Ag-HA coating groups. *Significant differences between the 50% Ag-HA coating group and the other two groups at 28 and 56 days (*P* < 0.01).

Mean silver concentrations in the kidneys for the HA, 2% Ag-HA, and 50% Ag-HA coating groups at 2 days were 0.03 ± 0.01 *μ*g/g, 0.01 ± 0.00 *μ*g/g, and 0.01 ± 0.00 *μ*g/g, respectively (Figure 5). At 3 days, mean silver concentrations for the HA, 2% Ag-HA, and 50% Ag-HA coating groups were 0.02 ± 0.01 *μ*g/g, 0.01 ± 0.00 *μ*g/g, and 0.02 ± 0.01 *μ*g/g, respectively. At 4 days, mean silver concentrations for the HA, 2% Ag-HA, and 50% Ag-HA coating groups were 0.02 ± 0.00 *μ*g/g, 0.01 ± 0.00 *μ*g/g, and 0.01 ± 0.00 *μ*g/g, respectively. No significant differences were found between the 2% Ag-HA and 50% Ag-HA coating groups at 2, 3, and 4 days.

The highest silver concentrations were found in the kidneys of the 50% Ag-HA coating group at 4 weeks, with a mean concentration of 0.04 ± 0.01 *μ*g/g. The concentration of Ag decreased gradually over the experimental period. Mean silver concentrations of the 50% Ag-HA coating groups at 8 and 12 weeks were 0.03 ± 0.01 *μ*g/g and 0.02 ± 0.01 *μ*g/g, respectively. Silver concentrations in HA and 2% Ag-HA coating groups were 0.01 ± 0.00 *μ*g/g at 4, 8, and 12 weeks. The value in the 50% Ag-HA coating group differed significantly from that in the HA and 2% Ag-HA coating groups at 4 and 8 weeks (*P* < 0.01 for all comparisons at 4 and 8 weeks, Tukey's HSD test). No significant differences were found between the three groups at 12 weeks.

#### 3.3.4. Spleen

**Figure 6:**
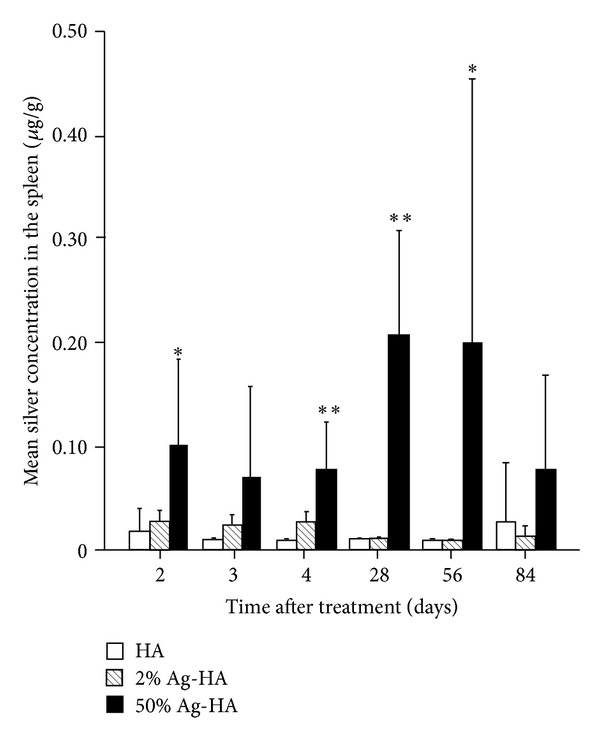
Mean silver concentration in the liver at 2, 3, 4, 28, 56, and 84 days after treatment in the hydroxyapatite (HA), 2% Ag-HA, and 50% Ag-HA coating groups. *Significant differences between the 50% Ag-HA coating group and the other two groups at 2 and 56 days (*P* < 0.05). **Significant differences between the 50% Ag-HA coating group and the other two groups at 4 and 28 days (*P* < 0.01).

Mean silver concentrations in the spleen for the HA, 2% Ag-HA, and 50% Ag-HA coating groups at 2 days were 0.01 ± 0.00 *μ*g/g, 0.03 ± 0.01 *μ*g/g, and 0.10 ± 0.04 *μ*g/g, respectively (Figure 6). At 3 days, mean silver concentrations for the HA, 2% Ag-HA, and 50% Ag-HA coating groups were 0.01 ± 0.00 *μ*g/g, 0.02 ± 0.01 *μ*g/g, and 0.07 ± 0.04 *μ*g/g, respectively. At 4 days, mean silver concentrations for the HA, 2% Ag-HA, and 50% Ag-HA coating groups were 0.01 ± 0.00 *μ*g/g, 0.03 ± 0.01 *μ*g/g, and 0.08 ± 0.02 *μ*g/g, respectively. The value in the 50% Ag-HA coating group differed significantly from that in the HA and 2% Ag-HA coating groups at 2 and 4 days (*P* < 0.05 for all comparisons at 2 days and *P* < 0.01 for all comparisons at 4 days, Tukey's HSD test). No significant differences were found between the HA and 2% Ag-HA coating groups at 2, 3, or 4 days.

The highest silver concentrations were found in the spleen for the 50% Ag-HA coating group at 4 weeks, with a mean concentration of 0.21 ± 0.05 *μ*g/g. The concentration of Ag decreased gradually over the experimental period. Mean silver concentration for the 50% Ag-HA coating group at 8 weeks was 0.20 ± 0.13 *μ*g/g. Silver concentration for the HA and 2% Ag-HA coating groups was 0.01 ± 0.00 *μ*g/g at 4 and 8 weeks. At 12 weeks, mean silver concentrations for the HA, 2% Ag-HA, and 50% Ag-HA coating groups were 0.03 ± 0.03 *μ*g/g, 0.01 ± 0.01 *μ*g/g, and 0.08 ± 0.05 *μ*g/g, respectively. The value in the 50% Ag-HA coating group differed significantly from those in the HA and 2% Ag-HA coating groups at 4 and 8 weeks (*P* < 0.01 for all comparisons at 4 weeks and *P* < 0.05 for all comparisons at 8 weeks, Tukey's HSD test). No significant differences were found between the three groups at 12 weeks.

### 3.4. Histological Examination

Although data are not shown, growth of fibrous tissue, chronic inflammatory infiltrates, foreign body granulomas, and fatty degeneration was not detected in the brain, liver, kidneys, or spleen of the HA, 2% Ag-HA, and 50% Ag-HA coating groups at any time during the experimental period. Histological examination of organs revealed no abnormal pathological findings.

## 4. Discussion

Silver has a broad antibacterial spectrum, strong antibacterial activity, and low toxicity. Devices such as heart valves [13], central venous catheters [14], aortic grafts [15], urethral catheters [16], burn wound dressings [17], and megaprostheses [18] have been coated with silver or silver compounds. Silver metal-coated megaprostheses have already been used in European countries and good clinical results have been reported [19, 20]. However, such silver metal coatings have not been applied to the surface of the stem, in the region of bone contact, because of potential toxicity. To resolve this problem, we used HA as a support material for silver ions, because it offers good biocompatibility and osteoconductivity. We previously reported that no cytotoxicity resulted from silver in the Ag-HA coating according to both extraction and direct methods* in vitro* [9]. However, no reports have described the* in vivo* toxicity of Ag-HA. Our results shown here suggest that no acute or subacute toxicity occurs* in vivo* with 2% Ag-HA coating as well as HA coating. This is the first report about the* in vivo* toxicity of Ag-HA.

Wan et al. reported that silver levels below 200 ppb in blood could be considered normal, because humans incorporate orally each day small amounts of silver [21]. Only small amounts ofsilver will be resorbed by the intestine and transported as a complex with plasma proteins. Most silver is then excreted by the liver. The rest of the silver is stored and accumulated intracellularly in organs and tissues without any use. Gaul and Staud estimated that an average 50-year-old man would store 0.2–1 g of silver [22]. Argyria, a gray-blue discoloration of a tissue, is most commonly observed in humans exposed to silver. This pathological finding was seen more commonly in the 19th century in association with occupational exposure in silversmiths, miners, and photographs [23, 24]. Argyria can also appear from the use of silver-containing medications. We showed in this study that no animals had any signs of local or systemic argyrosis. In the literature, no consistent data have been reported concerning the dose of silver necessary to cause argyria. Gaul and Staud revealed that a minimum amount of 1.8 g of silver is necessary to cause argyria [22]. The amount of silver in the 2% Ag-HA coating implant used in this study is about 15 *μ*g/cm^2^. The total amount of silver necessary for the 2% Ag-HA coating on femoral replacements in humans (prosthesis surface area of coating, 76 cm^2^) is, therefore, about 1.14 mg. The small amount of silver used here would not cause argyria.

A concentration of silver in the blood of more than 300 ppb has been reported to cause argyria, argyrosis, and liver and kidney damage [25]. Drake and Hazelwood found that acute symptoms of overexposure to silver nitrate include a decrease in blood pressure, diarrhea, irritation of the stomach, and decreased respiration [26]. Chronic symptoms resulting from intake of a low dose of silver salts are fatty degeneration in the liver and kidneys [26]. Long-term inhalation or ingestion of soluble silver compounds or colloidal silver may cause argyria and/or argyrosis. In our study, the highest concentrations of serum silver resulting from the 2% Ag-HA and 50% Ag-HA coatings were 1.75 ± 1.08 ppb at 2 days and 16.2 ± 5.85 ppb at 3 days, respectively. These levels were low enough to avoid harmful effects. Histological examination also revealed that fatty degeneration was not detected in the liver or kidneys of the HA, 2% Ag-HA, and 50% Ag-HA coating groups in the subacute phase. Mean values of GOT, GPT, LDH, creatinine, and BUN showed no significant differences between control, 2% Ag-HA, and 50% Ag-HA coating groups in any experimental periods. Although mean GOT and LDH levels showed significant differences between the control and HA coating groups at 4 and 8 weeks, this would be due to hemolysis, because the GPT level in the HA coating group was not elevated compared to the control group.

In this study, mean silver concentrations in all analyzed organs of the 2% Ag-HA coating group showed no significant differences compared with those in the HA coating group at all experimental periods. The silver concentration of 50% Ag-HA was significantly elevated in all analyzed organs. The highest silver concentrations were found in the brain, liver, kidneys, and spleen of the 50% Ag-HA coating group at 4 weeks, with mean concentrations of 0.05 *μ*g/g, 0.05 *μ*g/g, 0.04 *μ*g/g, and 0.21 *μ*g/g, respectively. The concentration of silver decreased gradually over the experimental period, and no significant differences were found between the three groups at 12 weeks, with neither pathological changes in laboratory parameters nor histological changes in tissues. We reported in a previous study that silver toxicity depends on the dose [12]. Although the silver concentration in the blood was the highest in the acute phase and silver concentrations of organs peaked in the subacute phase in this study, no silver toxicity was encountered during either period. Although no data were obtained regarding chronic symptoms resulting from the implantation of the Ag-HA coating after 12 weeks, most silver would be gradually excreted. Silver toxicity such as systemic argyrosis, damage, and fatty degeneration of the liver and kidneys may thus be unlikely. In the future, it will be necessary to implant Ag-HA-coated megaprostheses in humans to assess possible toxicity and to determine infection rates with joint replacement.

## 5. Conclusions

We have shown that the use of 2% and 50% Ag-HA-coated implants is associated with neither acute nor subacute toxicity in the rat tibia. However, data about chronic toxicity are still missing. Since the 50% Ag-HA coating inhibits bone formation [12], silver concentrations of Ag_2_O powder mixed with HA powder of around 2-3% appear appropriate. Ag-HA coating on an implant may offer a biologically safe antibacterial biomaterial and may be of value in reducing SSI related to implantation.

## Figures and Tables

**Table 1:** Laboratory parameters in the acute phase (2–4 days postoperatively).

Time after treatment	Implant coating	GOT (IU/L)	GPT (IU/L)	LDH (IU/L)	Creatinine (mg/dL)	BUN (mg/dL)
	Control	73.0 ± 17.3	31.0 ± 3.0	851.7 ± 458.1	0.22 ± 0.01	21.1 ± 1.1
2 days	HA	80.3 ± 19.2	30.3 ± 1.5	1316.0 ± 569.5	0.22 ± 0.03	18.6 ± 1.6
	2% AgHA	84.6 ± 9.1	34.0 ± 5.1	1018.0 ± 292.5	0.23 ± 0.01	21.4 ± 1.9
	50% AgHA	67.7 ± 8.3	26.7 ± 1.2	730.3 ± 243.1	0.22 ± 0.02	20.7 ± 2.0
3 days	HA	50.0 ± 7.0	25.7 ± 2.1	394.7 ± 121.6	0.25 ± 0.02	22.0 ± 4.9
	2% AgHA	75.0 ± 6.2	34.7 ± 1.5	681.3 ± 188.6	0.26 ± 0.01	21.4 ± 0.4
	50% AgHA	63.3 ± 2.1	26.7 ± 3.5	562.7 ± 64.9	0.21 ± 0.02	19.4 ± 0.6
4 days	HA	87.3 ± 8.7	31.3 ± 1.2	1125.0 ± 147.8	0.20 ± 0.01	17.8 ± 0.4
	2% AgHA	61.3 ± 11.6	27.0 ± 0.0	409.7 ± 235.3	0.25 ± 0.02	17.6 ± 0.7
	50% AgHA	64.3 ± 3.1	26.0 ± 1.7	725.0 ± 251.2	0.22 ± 0.02	17.9 ± 1.1

Five 10-week-old male Sprague-Dawley rats with no treatment were used as controls. No significant differences were seen between the control group and all comparators.

GOT: glutamic-oxaloacetic transaminase; GPT: glutamic-pyruvic transaminase; LDH: lactate dehydrogenase; BUN: blood urea nitrogen.

**Table 2:** Laboratory parameters in the subacute phase (4–12 weeks postoperatively).

Time after treatment	Implant coating	GOT (IU/L)	GPT (IU/L)	LDH (IU/L)	Creatinine (mg/dL)	BUN (mg/dL)
4 weeks	Control (14-week-old)	72.7 ± 7.1	34.0 ± 2.6	820.3 ± 267.8	0.25 ± 0.02	20.8 ± 0.4
	HA	95.6 ± 12.9*	31.6 ± 1.2	1180.3 ± 475.6	0.27 ± 0.01	22.1 ± 1.4
	2% AgHA	82.7 ± 5.1	34.7 ± 1.2	884.7 ± 163.1	0.25 ± 0.02	22.3 ± 0.8
	50% AgHA	73.7 ± 7.5	34.3 ± 2.1	619.3 ± 332.6	0.27 ± 0.02	21.3 ± 1.2

8 weeks	Control (18-week-old)	80.4 ± 37.6	35.6 ± 5.0	836.2 ± 904.7	0.28 ± 0.05	21.6 ± 2.5
	HA	131.0 ± 20.1**	38.2 ± 3.4	2202.2 ± 824.6***	0.31 ± 0.03	23.2 ± 4.2
	2% AgHA	102.0 ± 5.1	40.6 ± 5.2	1562.8 ± 278.4	0.33 ± 0.02	23.5 ± 1.9
	50% AgHA	78.4 ± 14.8	36.8 ± 2.7	705.2 ± 304.6	0.31 ± 0.01	23.5 ± 1.7

12 weeks	Control (22-week-old)	99.7 ± 8.1	33.3 ± 2.3	1649.0 ± 306.2	0.31 ± 0.03	19.6 ± 2.1
	HA	90.5 ± 9.2	33.5 ± 4.9	1799.0 ± 429.9	0.31 ± 0.01	20.3 ± 2.2
	2% AgHA	101.8 ± 12.5	36.0 ± 3.4	1895.0 ± 527.2	0.31 ± 0.01	22.2 ± 1.0
	50% AgHA	82.3 ± 2.5	32.3 ± 2.1	1367.7 ± 157.1	0.28 ± 0.01	21.7 ± 1.5

Five 14-, 18-, and 22-week-old male Sprague-Dawley rats with no treatment were used as controls for 4, 8, and 12 weeks, respectively. *Mean GOT levels at 4 weeks differed significantly between controls (14-week-old rats) and the HA group (*P* < 0.05). ** and ***are the mean GOT and LDH levels at 8 weeks that differed significantly between controls (18-week-old rats) and the HA group (*P* < 0.05).

GOT: glutamic-oxaloacetic transaminase; GPT: glutamic-pyruvic transaminase; LDH: lactate dehydrogenase; BUN: blood urea nitrogen.
